# Physician-Perceived Barriers to Healthcare Transition for Young Adults With Special Healthcare Needs

**DOI:** 10.7759/cureus.101473

**Published:** 2026-01-13

**Authors:** Ruchita Iyer, Jinia Chakraborty, Emily N Bufkin

**Affiliations:** 1 Internal Medicine - Pediatrics, University of Texas Southwestern Medical Center, Dallas, USA

**Keywords:** children and youth with special healthcare needs, complex care, healthcare transition, needs assessment, quality improvement (qi)

## Abstract

Introduction

Healthcare transition (HCT), or the process of moving from pediatric to adult care, can be a complex process requiring care coordination and communication, especially for young adults with special healthcare needs (YASHCN). Limited access to adult-system resources such as care coordination and social work can result in poorly facilitated HCT, leading to increased healthcare expenditures, medical complications, and loss to follow-up.

Methods

We conducted mixed-methods interviews incorporating structured surveys with general and subspecialty adult physicians at the University of Texas Southwestern Medical Center, a large academic medical center in Dallas, Texas. Physicians were asked to identify major, minor, or absent barriers encountered when receiving YASHCN and to describe potential solutions at both specialty-specific and system-wide levels. Quantitative responses were summarized as counts and percentages, and exact binomial confidence intervals were calculated for the proportions endorsing each barrier as major or minor. Exact binomial tests assessed whether endorsement exceeded 50%. Qualitative responses were analyzed using thematic analysis.

Results

Physicians reported numerous barriers to HCT, with endorsement rates ranging from 40% to 91.7%. The most frequently identified barriers included lack of residency or fellowship training in YASHCN care (91.7%), poor patient or parent preparation for the adult model of care (86.7%), inadequate care-coordination support (86.7%), limited access to adult primary care physicians (84.6%), and lack of relevant clinical guidelines (80%). Several of these were endorsed at rates exceeding 50%, indicating strong physician consensus. Additional commonly reported barriers included poor communication during provider transfers (67.7%), limited access to subspecialists (67.7%), patient or parent mistrust of adult care (75%), insufficient social work support (60%), limited time to complete paperwork (60%), difficulty accessing pediatric medical records (53.3%), limited access to needed specialty services (40%), and physicians’ own limited knowledge of chronic childhood-onset diseases (46.7%). When asked to elaborate on these perceived barriers and identify potential solutions, physicians emphasized the need for better patient and caregiver education, stronger communication between pediatric and adult teams to improve access to medical records, building trust in the adult model of care, and system-level supports such as increased care coordination and social work support, standardized communication processes, and increased institutional investment in HCT services.

Conclusion

These physician-identified findings, supported by existing literature, highlight the importance of implementing collaborative solutions to bridge pediatric and adult care for this vulnerable patient population. These findings also highlight the importance of targeted educational efforts to improve clinician preparedness in caring for YASHCN. This stakeholder-driven approach informs the development of an evidence-based, centralized HCT clinic, serving as a crucial first step to improve HCT readiness in YASCHN and, in turn, improve health outcomes and quality of life for this vulnerable population.

## Introduction

The process of transitioning from pediatric to adult models of care, known as a “healthcare transition” (HCT), begins around 12 years of age and continues through 21 years [1]. The goal of this process is to increase patient self-management in care. Addressing HCT requires coordination between the pediatric care team, the patient and their family, and the adult care team.  

HCT often presents unique challenges for patients and healthcare systems. A systematic review assessing quality indicators in HCT found that there are no standardized guidelines or universal approach to guide the care of chronic childhood-onset diseases (CCOD) [2]. Furthermore, adult physicians report feeling unprepared or not adequately trained to care for adult patients with CCOD [3]. In addition to medical care, pediatric care models and support systems also differ from adult models. Pediatric models of care involve parental decision-making and interdisciplinary support, such as multidisciplinary and complex care clinic models for chronic medical conditions. The adult model of care assumes patient autonomy to not only make medical decisions but also to navigate the health system [4]. Per the 2023 National Survey of Children's Health, only 21.9% of children reported receiving services for transition to adult care, defined as care meeting all three HCT core elements: (1) individual time with their provider in a preventative care visit, (2) collaboration with their physician to learn self-care and self-efficacy skills to navigate their transition, and (3) specific discussion regarding future adult-provider care) [5].  With these challenges in mind, it comes as little surprise that children who do not receive adequate transition care support face increased risk of long-term adverse health outcomes, health complications, increased healthcare costs, and decreased quality of life [6].

Young adults with special healthcare needs (YASHCN), defined as patients with chronic health conditions that require three or more subspecialty needs, require greater care coordination and support to bridge their care. Thus, these patients are at greater risk of adverse health outcomes with poorly facilitated transitions [7-9]. Studies have shown that YASHCN who do not receive transition care and services face adverse health outcomes and greater healthcare expenditures [6, 10]. Given the increased risk of poor health outcomes and greater healthcare utilization, combined with the poorly reported receipt of HCT services, this vulnerable time period offers much room for improvement.

Part of improving HCT is a better understanding of the system-wide barriers and challenges when preparing and receiving patients. Physician-perceived barriers to successful HCT have been studied in the past, including communication/consultation gap, training limitations, care delivery, such as lack of care coordination, lack of patient knowledge/engagement, lack of comfort/familiarity with adult model of care, patient/caregiver family dynamics, such as reluctance to relinquish responsibility [11].

To better understand the needs of adult physicians at a large academic medical center with affiliated county, university, and children’s hospitals, we conducted a mixed-methods needs assessment to inform the development of an institution-wide, consultative transition services clinic. The primary objective was to identify physician-perceived barriers to receiving and providing care for patients with CCOD, while secondary objectives were to characterize the strength of endorsement of specific barriers and to explore physician-identified, system-level, and specialty-specific solutions. This assessment represents an initial step toward identifying barriers encountered by adult physicians during care transfer and guiding the development of institutional resources to support patients and clinicians throughout the HCT process and subsequent integration into adult care.

## Materials and methods

This is a mixed quantitative/qualitative descriptive survey of adult physicians conducted at the University of Texas Southwestern Medical Center, a large academic medical center in Dallas, Texas. This project was reviewed by the UT Southwestern Human Research Protection Program (HRPP) and determined to be non-regulated research. The research team consisted of the senior author, who is a female assistant professor of internal medicine and pediatrics at a large academic medical center, and two medical students.

The senior author contacted division directors in General and Subspecialty Internal Medicine, Neurology, Urology, Physical Medicine and Rehabilitation (PM&R), and Psychiatry to identify faculty members who provide care for young adults with special healthcare needs (YASHCN). Specialties outside of Internal Medicine were selected based on their higher likelihood of caring for patients with childhood-onset disabilities (CCOD), such as Urology for young adults with spina bifida and PM&R for individuals with cerebral palsy. Identified physicians were invited via email to participate in a virtual interview regarding the current state of HCT at a large academic medical center. Participation was voluntary, and no incentives were provided. The study used a convenience sample of physicians who agreed to participate during the study period. A total of 27 physicians were invited, and 15 agreed to participate. Thus, the sample size was determined entirely by the number of available respondents rather than by an a priori statistical power calculation. Given this design and the exploratory, mixed-methods nature of the project, a post hoc power analysis was not conducted, and quantitative findings are interpreted cautiously, with emphasis on descriptive estimates and confidence intervals rather than confirmatory inference.

The senior author conducted 30-minute virtual interviews with respondents using Microsoft Teams. Each interview was recorded and transcribed by an observing member of the research team. During the interview, physicians were asked questions from a standardized survey that included both quantitative and qualitative components. The complete survey instrument is provided in the Appendix. The participants were asked whether they perceived the following items as a major barrier, minor barrier, or not a barrier to receiving YASHCN patients: difficulty accessing pediatric medical records; poor communication during transfer from pediatric providers; lack of access to the patient’s adult primary care physician; lack of access to other pediatric or adult subspecialty physicians involved in care; lack of access to needed specialty services; time required to complete necessary paperwork or forms; poor patient or parent preparation for the adult model of care; patient or parent mistrust of new adult providers or the adult healthcare system; availability of social work support; availability of care coordination support; physician knowledge of pediatric-onset medical conditions; availability of clinical guidelines for the care of YASHCN; and residency or fellowship training in the care of YASHCN. These pre-selected barriers were adapted from previously published data on physician-perceived barriers [11]. Quantitative responses were entered into REDCap, a secure online database. If a participant was unable to provide a single response to a Likert-scale item, no response was entered for that item. Qualitative responses were entered into the database using the automatically generated interview transcript.

Quantitative survey responses were summarized using counts and percentages. For each of the 13 predefined barrier items, the proportion of physicians endorsing the item as a major or minor barrier was calculated. Exact 95% confidence intervals were generated using the Clopper-Pearson method. Exact two-sided binomial tests were performed to evaluate whether the observed proportion exceeded a neutral reference value of 0.50, representing equal likelihood of endorsement versus non-endorsement of a given barrier. Barriers with statistically significant p-values were interpreted as demonstrating physician consensus exceeding chance agreement. Given the exploratory design and small sample size, quantitative findings are presented with emphasis on proportions and confidence intervals rather than statistical significance alone.

Qualitative data were analyzed using the Braun and Clarke model of thematic analysis [12]. Initial coding was conducted by the first author using an inductive approach, with codes developed iteratively through review of deidentified transcripts of qualitative responses by question. Codes were subsequently organized into subthemes and overarching themes through discussion within the primary research team. A second research team member, who was not involved in the initial coding or theme development, independently reviewed the coding framework and thematic structure, with any discrepancies resolved through consensus.

## Results

Fifteen faculty representatives from the Internal Medicine divisions, Neurology, Psychiatry, Physical Medicine and Rehabilitation, and Urology departments participated in this study (Table 1). Sixty percent (n = 9) were male, and 40% (n = 6) were female. The average number of years post-clinical training was 12.77 years (range of 1-25 years). 

**Table 1:** Participant demographics Average years of post-training clinical practice = 12.73

Participant ID	Specialty	Years of post-training clinical practice
1	Pulmonary Medicine	14
2	Cardiology - Adult congenital heart disease	1
3	Genetics	5
4	Pulmonary Medicine	5
5	General Internal Medicine	12
6	Neurology	16
7	Psychiatry	3
8	Cardiology	18
9	Urology	23
10	Hematology/Oncology - malignant hematology and stem cell transplant	23
11	Neurology	25
12	Endocrine	7
13	General Internal Medicine	14
14	Gastroenterology	15
15	Infectious Disease	10

Quantitative results

Table 2 summarizes the predefined aspects of HCT that physicians identified as major, minor, or not a barrier. To further characterize the strength of physician agreement, exact binomial confidence intervals and tests comparing endorsement rates to a neutral reference value of 0.50 were calculated for each barrier item (Table 3). Across the 15 participating physicians, endorsement of barriers as major or minor ranged from 40% to 91.7%.

**Table 2:** Physician-endorsed healthcare transition barriers at a large academic medical center *Abbreviations:* SHCN: special healthcare needs; YASHCN: young adults with special healthcare needs

	Difficulty accessing pediatric medical records	Poor communication when transferring pediatric providers	Lack of access to patient's adult primary care physician	Lack of access to other pediatric or adult subspecialty physicians involved in care	Lack of access to needed specialty services (physical/occupational/speech/nutrition services)	Time needed to complete the necessary paperwork/forms for patients with SHCN	Poor patient or parent preparation for the adult model of care	Patient or parent mistrust of new adult providers or the adult healthcare system	Not enough social work support	Not enough care coordinator support	My own lack of knowledge about pediatric-onset medical conditions	Lack of existing clinical guidelines for the care of young adults with pediatric-onset SHCN	Lack of residency/ fellowship training in the care of YASHCN
Major barrier	4 (26.67%)	4 (26.67%)	3 (23.07%)	2 (13.33%)	1 (6.67%)	6 (40.00%)	7 (46.67%)	6 (50.00%)	6 (40.00%)	9 (60.00%)	3 (20.00%)	4 (26.67%)	7 (58.33%)
Minor barrier	4 (26.67%)	6 (40.00%)	8 (61.54%)	8 (53.33%)	5 (33.33%)	3 (20.00%)	6 (40.00%)	3 (25.00%)	3 (20.00%)	4 (26.67%)	4 (26.67%)	8 (53.33%)	4 (33.33%)
Not a barrier	7 (46.67)	5 (33.33%)	2 (15.39%)	5 (33.33%)	9 (60.00%)	6 (40.00%)	2 (13.33%)	3 (25.00%)	6 (40.00%)	2 (13.33%)	8 (53.33%)	3 (20.00%)	1 (8.33%)
Total	15	15	13	15	15	15	15	12	15	15	15	15	12

**Table 3:** Exact binomial analysis of physician-endorsed healthcare transition barriers at a large academic medical center Proportions represent the number of physicians endorsing each item as a major or minor barrier. P-values were calculated using exact two-sided one-sample binomial tests comparing observed endorsement proportions to a neutral reference value of p₀ = 0.50, representing equal likelihood of endorsement versus non-endorsement. The binomial test statistic (k) denotes the number of physicians endorsing each barrier out of the total number responding to that item (N). No between-group comparisons were performed. Statistical significance was defined as p ≤ 0.05. Exact 95% confidence intervals were calculated using the Clopper–Pearson method. *Abbreviations*: k: number endorsing barrier; N: total responses; CI: confidence interval; YASHCN: young adults with special healthcare needs; CCOD: chronic childhood-onset diseases

Barrier item	k / N endorsing barrier	Proportion (%)	95% CI (Exact)	Binomial test statistic (k)	p-value
Lack of residency/fellowship training in YASHCN care	11 / 12	91.7	0.62–0.998	11	0.006*
Poor patient or parent preparation for the adult model of care	13 / 15	86.7	0.60–0.98	13	0.007*
Insufficient care-coordination support	13 / 15	86.7	0.60–0.98	13	0.007*
Lack of access to an adult primary care physician	11 / 13	84.6	0.55–0.98	11	0.022*
Lack of clinical guidelines for YASHCN	12 / 15	80.0	0.52–0.96	12	0.035*
Poor communication during transfer from pediatric providers	10 / 15	66.7	0.38–0.88	10	0.30
Lack of access to other pediatric or adult subspecialists	10 / 15	66.7	0.38–0.88	10	0.30
Patient or parent mistrust of the adult healthcare system	9 / 12	75.0	0.43–0.95	9	0.146
Time required to complete paperwork/forms	9 / 15	60.0	0.32–0.84	9	0.61
Insufficient social work support	9 / 15	60.0	0.32–0.84	9	0.61
Difficulty accessing pediatric medical records	8 / 15	53.3	0.27–0.79	8	1.00
Physician's lack of knowledge of CCOD	7 / 15	46.7	0.21–0.73	7	1.00
Lack of access to needed specialty services	6 / 15	40.0	0.16–0.68	6	0.61

Difficulty accessing pediatric medical records was identified as a barrier by eight physicians (53.3%), while poor communication during transfer from pediatric providers was identified by 10 physicians (67.7%). Eleven physicians (84.6%) identified lack of access to a patient’s adult primary care physician as a barrier, and 10 (67.7%) reported limited access to other pediatric or adult subspecialty physicians involved in care. Six physicians (40%) identified lack of access to needed specialty services, including physical, occupational, speech, or nutrition services, as a barrier. Nine physicians (60%) reported that the time required to complete necessary paperwork or forms represented a barrier.

Barriers related to patient and system readiness were frequently endorsed. Thirteen physicians (86.7%) identified poor patient or parent preparation for the adult model of care as a major or minor barrier, and nine (75%) identified patient or parent mistrust of new adult providers or the adult healthcare system as a barrier. Insufficient social work support was identified by nine physicians (60%), while inadequate care-coordination support was identified by 13 physicians (86.7%). Knowledge- and training-related barriers were also common: seven physicians (46.7%) identified their own limited knowledge of chronic childhood-onset diseases as a barrier, 12 (80%) reported a lack of existing clinical guidelines for the care of YASHCN, and 11 (91.7%) identified insufficient residency or fellowship training in caring for YASHCN.

Exact binomial testing demonstrated that endorsement of several barriers, including lack of residency or fellowship training, poor patient or parent preparation for adult care, insufficient care-coordination support, limited access to adult primary care physicians, and lack of clinical guidelines, exceeded the neutral reference threshold, indicating substantial physician agreement. For the remaining barriers, confidence intervals were wider and included the reference value, reflecting greater variability in physician endorsement.

Qualitative results: physician-reported barriers to receiving YASHCN

Participants who identified the predefined potential barriers as major barriers to receiving young adults with special healthcare needs were then asked to elaborate further. A research team member recorded these responses and analyzed them using thematic analysis (Table 4). 

**Table 4:** Thematic analysis of perceived major barriers when receiving young adults with special healthcare needs (YASHCN)

Major barrier	Quotes
Patient mistrust	Participant ID 3: "They're very mistrustful that nobody's going to understand their disease. You know, they're not going to call the right people. So that's definitely been a recurring theme in multiple families with inborn errors."
Patient preparation	Participant ID 2: "Our transition process is terrible, just terrible, and I know this because we're trying to improve it from the children's side of things. But yeah, kids aren't prepared, and adults don't know.”
Accessing medical records	Participant ID 9: "Every time I've tried to get anything from [the children’s hospital], it's.. at least 10-15 minutes to try to find it. If I do find it, most often I don't find it, so I'll end up just calling one of my colleagues to try to fill me in. So I just feel like that could be it may be possible. It just could be extremely streamlined somehow..."
Establishing care	Participant ID 4: “That's my biggest barrier that we that's the biggest barrier we face is getting those patients plugged in and set up, and then that is very unfortunately mostly insurance, and then occasionally location dependent.”
Lack of social work/care coordination support	Participant ID 2: “You need more backup, you need more. We need more phone lines. We need more secretaries. We need more front office…we never need more administrators. But we need more people who can work, sort of the backlines, so that the doctors and nurse practitioners and people can just take care of patients without dealing with all the paperwork.”

Physician-reported challenges to receiving YASHCN 

**Figure 1:**
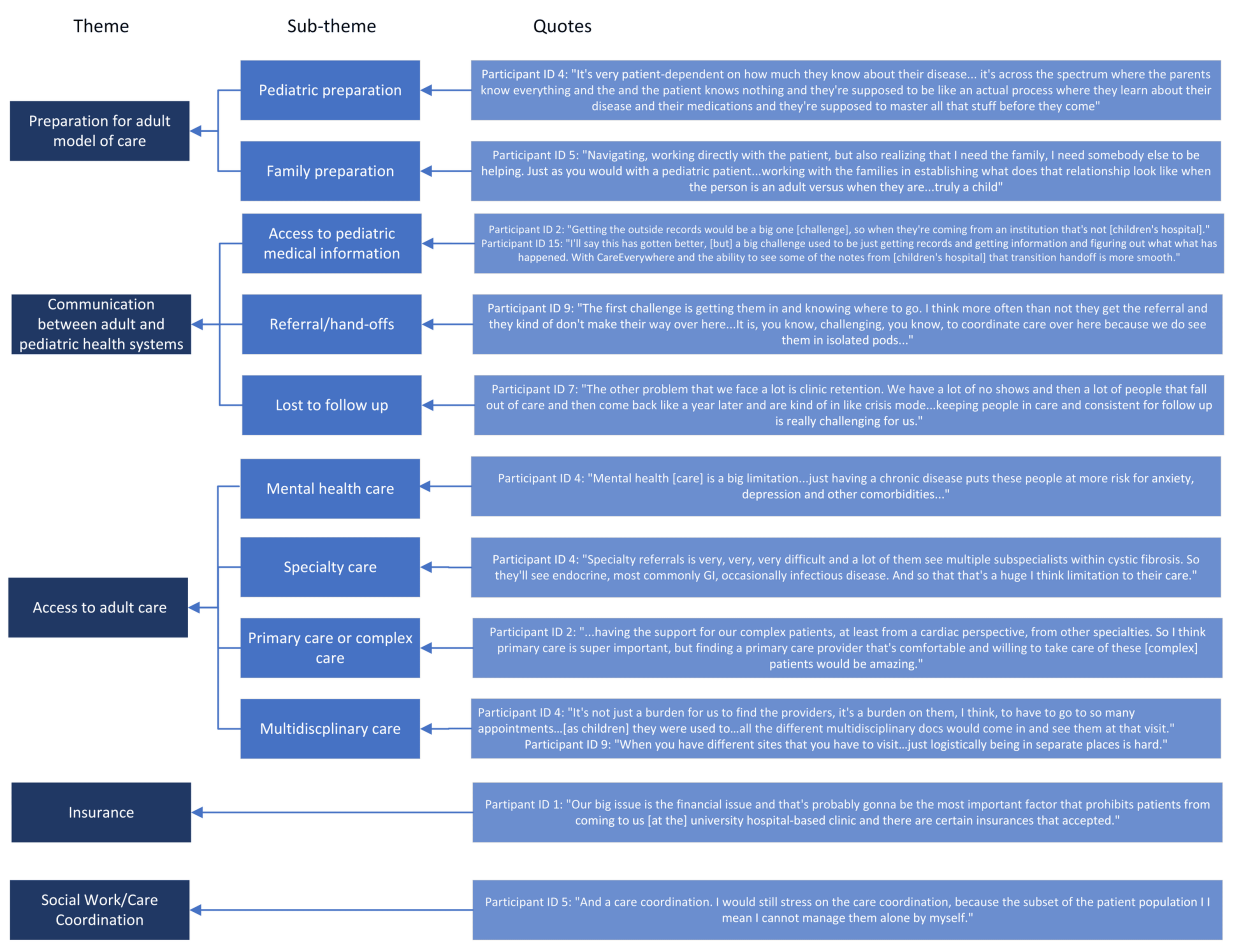
Themes and subthemes identified as challenges to receiving young adults with special healthcare needs Image credits: Ruchita Iyer, MD via Microsoft Visio (Microcsoft Corp., USA)

When asked about system-wide and subspeciality specific challenges, physicians identified (1) access to social work or care coordination, (2) insurance, (3) access to adult primary care or complex care clinics, (4) lack of multidisciplinary clinics, (5) challenges with the referral process and accessing medical information, (6) poor preparation for adult model of care, and (7) working with families/patients with limited ability to communicate (Figure 1).

Lack of Social Work and Care Coordination Support

Physicians noted inadequate access to social work and care coordination as a challenge to receiving YASHCN. These patients often require durable medical equipment and other resources, requiring paperwork completion to ensure continued access to services. Several participants commented on the amount of paperwork completed when receiving patients with complex conditions. Those physicians, such as participant ID 1, who had social work or care coordinator support embedded in their clinical practice, even pointed out “shortages. Our nurses try to help, but we don't have as much support to complete paperwork."

Insurance

Many physicians commented that differences between pediatric and adult public insurance (such as Medicaid) pose a challenge for receiving YASHCN. When transitioning from pediatric to adult insurance models, some patients lose coverage for previously covered services, while others may not have adequate insurance to see the recommended adult specialists. In addition to different benefits and available coverage, participant ID 3 also commented on the different billing structures between pediatric and adult clinics: “...in most of the clinics, a nutritionist is integrated on the children's side. But on the adult side, it's a separate appointment. It's billed separately. Insurance only covers a couple of indications, and typically, it's not why they need to see them."

Inadequate Access to Adult Primary Care

Several specialty physicians commented on the lack of adequate access to primary care as a challenge in receiving YASHCN transitioning from pediatric to adult care. While patients may transfer and continue care with their primary specialist, such as a pulmonologist for cystic fibrosis, they may fail to transfer to adult primary care physicians, resulting in gaps in preventive care. One physician also noted that many YASHCN often transition from a complex care clinic, where they receive a higher level of support and resources than is typically available in adult primary care. With a lack of adult complex care clinics that provide a similar level of multidisciplinary support, offering primary care support to these patients can become a challenge to a successful HCT.

Lack of Multidisciplinary Clinics

Several physicians recognized the lack of adult multidisciplinary clinic models, increasing the transportation burden on patients and their families traveling between multiple appointments. They also felt that the lack of multidisciplinary clinics worsened communication and collaboration between different specialty providers.

Decentralized Referral Process and Difficulty Accessing Medical Information

Several physicians commented on challenges accessing pediatric medical records, especially when patients transfer from an external facility. While some physicians could remedy the lack of access by speaking with the referring pediatrician, several physicians reported receiving limited hand-offs, leaving patients and their families frustrated with the accepting physician’s apparent lack of knowledge about their condition and medical history. In addition to difficulty accessing medical information, physicians also reported challenges tracking referrals for high-risk patients, leading to loss to follow-up.

Poor Preparation for the Adult Model of Care

Physicians reported that patients transitioning to an adult model of care were often unprepared for the increased responsibility that the adult healthcare system demands. Some physicians felt that the transitioning YASHCN had inadequate medical knowledge of their condition(s), while others commented on challenges faced when working with parents or caregivers who were highly involved with and unlikely to relinquish control of their child’s care. A neurologist (participant ID 6) who works with patients with neurodevelopmental conditions noted that their patients’ limited ability to communicate posed additional challenges to their full integration into adult care.

Physician-reported potential solutions to receiving YASHCN 

**Figure 2:**
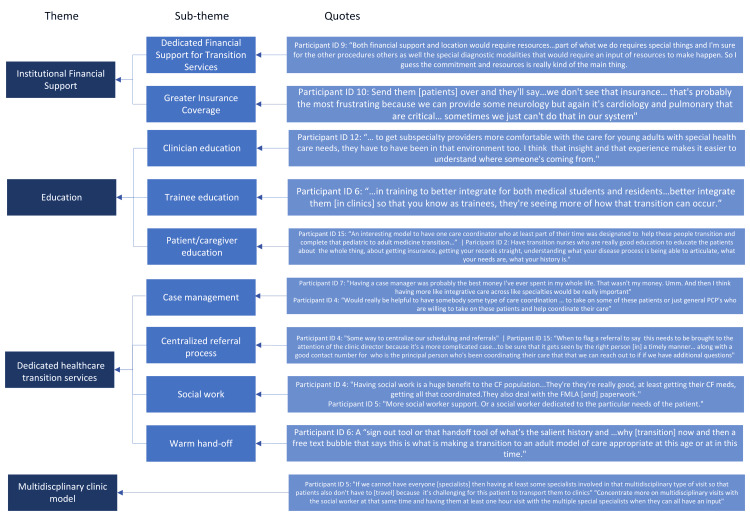
Themes and subthemes identified as solutions to receiving young adults with special healthcare needs Image credits: Ruchita Iyer, MD via Microsoft Visio (Microsoft Corp., USA)

When asked about potential solutions to perceived barriers and challenges to receiving patients at the system and subspeciality level, physicians suggested ideas that can be categorized mainly under three mains themes: (1) dedicated transition clinic resources that includes patient and caregiver education and care coordination; (2) institutional support for better financial resources and improved communication between transferring and accepting physicians; and (3) further medical training on YASHCN (Figure 2).

Dedicated Transition Clinic Resources

Many physicians commented on the need for a centralized process to facilitate better receipt and integration of YASCHN into the adult model of care. Suggested resources included a centralized referral process, identification of high-need referrals for earlier clinic appointments to reduce lost to follow-up, dedicated nurse educators to increase patient preparedness for HCT, and additional care coordination support to ensure patients have adequate healthcare access to address their medical needs and streamline the appointment process. Altogether, these solutions indicated the need for dedicated transition resources.

Institutional Support

Potential solutions to financial barriers include standardizing accepted insurance plans across the health system and providing adequate social work support for patients who lost insurance coverage during their HCT. Several physicians also commented on the need for institutional support beyond insurance reimbursement to support this vulnerable population. In addition to financial support, physicians noted that improved communication infrastructure across health systems would facilitate easier conversations between pediatric and adult physicians, enabling the accepting physician to better understand the medical history and needs of transferring patients.

Multidisciplinary Clinic Model

In addition to dedicated transition clinic resources, several physicians suggested creating multidisciplinary clinics to provide coordinated care for YASHCN requiring multiple subspecialty services. Inclusion of social work support and care coordination within these dedicated multidisciplinary clinics would provide the additional support, such as DME paperwork completion and insurance navigation, that physicians need to successfully integrate these medically complex patients into the adult system.

Further Medical Training

Lastly, physicians recognized the lack of HCT training received during their own medical training and offered that exposure to chronic childhood-onset diseases across multiple stages of training, from medical school to post-residency training, may not only better educate future physicians but also increase interest in this growing field.

## Discussion

In this study, we conducted a mixed quantitative/qualitative needs assessment of adult physicians to better understand the challenges and barriers to receiving pediatric patients with special healthcare needs. We identified multiple challenges to receiving YASHCN, with the strongest physician consensus centered on inadequate care coordination, poor patient or parent preparation for the adult model of care, limited access to adult primary care, and insufficient training and clinical guidance, alongside additional system- and resource-related barriers. These barriers can pose significant challenges to the successful transfer and integration of YASHCN into the adult model of care, with broader implications for effective care delivery, health outcomes, and the patient experience. It is notable, however, that physicians whose clinics had sufficient social work support did not comment on the lack of social work as a barrier to receiving patients. Instead, they often acknowledged how this resource was instrumental in facilitating the transition of and ongoing care of patients with complex care needs. This suggests that adequate social work resources not only play a critical role in the receipt of YASHCN but also emphasize the value of investing in this support.

These findings largely align with prior studies that interviewed patients and physicians, which identified patient access to care, financial constraints and insurance barriers, hesitancy to transition, and multidisciplinary system inefficiencies as challenges [13,14]. These findings highlight the continued need for meaningful change, including within our institution. Other challenges physicians shared in this needs assessment included access to medical records, while lack of physician training and clinical guidance emerged as highly endorsed barriers. 

Although mistrust in adult physicians was not among the most highly endorsed barriers in this needs assessment, prior work has identified trust as both a major barrier and a key facilitator of successful HCT [15]. Consistent with this literature, many of the solutions proposed by physicians in our study focused on mechanisms that foster trust, including improved communication between pediatric and adult care teams, dedicated transition services with care coordination, patient education, and standardized referral processes. While our study did not explicitly distinguish barriers from facilitators, the proposed solutions emphasized building trust not only in the transition process but also in the adult model of care and care teams. Additional solutions included multidisciplinary clinic models for patients with complex medical conditions, expanded institutional acceptance of public insurance plans and financial support, and improved training experiences related to childhood-onset diseases during residency and fellowship.

These proposed solutions closely align with existing evidence demonstrating that formalized transition processes supported by patient education and standardized timelines facilitate successful HCT [13,14]. Prior studies have shown that dedicated transition services improve successful transfers, self-management skills, and patient satisfaction across specialty clinics, including rheumatology, cystic fibrosis, and cerebral palsy [16-18]. In addition, care coordination has been identified by both physicians and patients as a central facilitator of successful HCT [15], further supporting efforts to develop a dedicated transition clinic staffed by physicians and ancillary personnel with institutional support.

**Figure 3:**
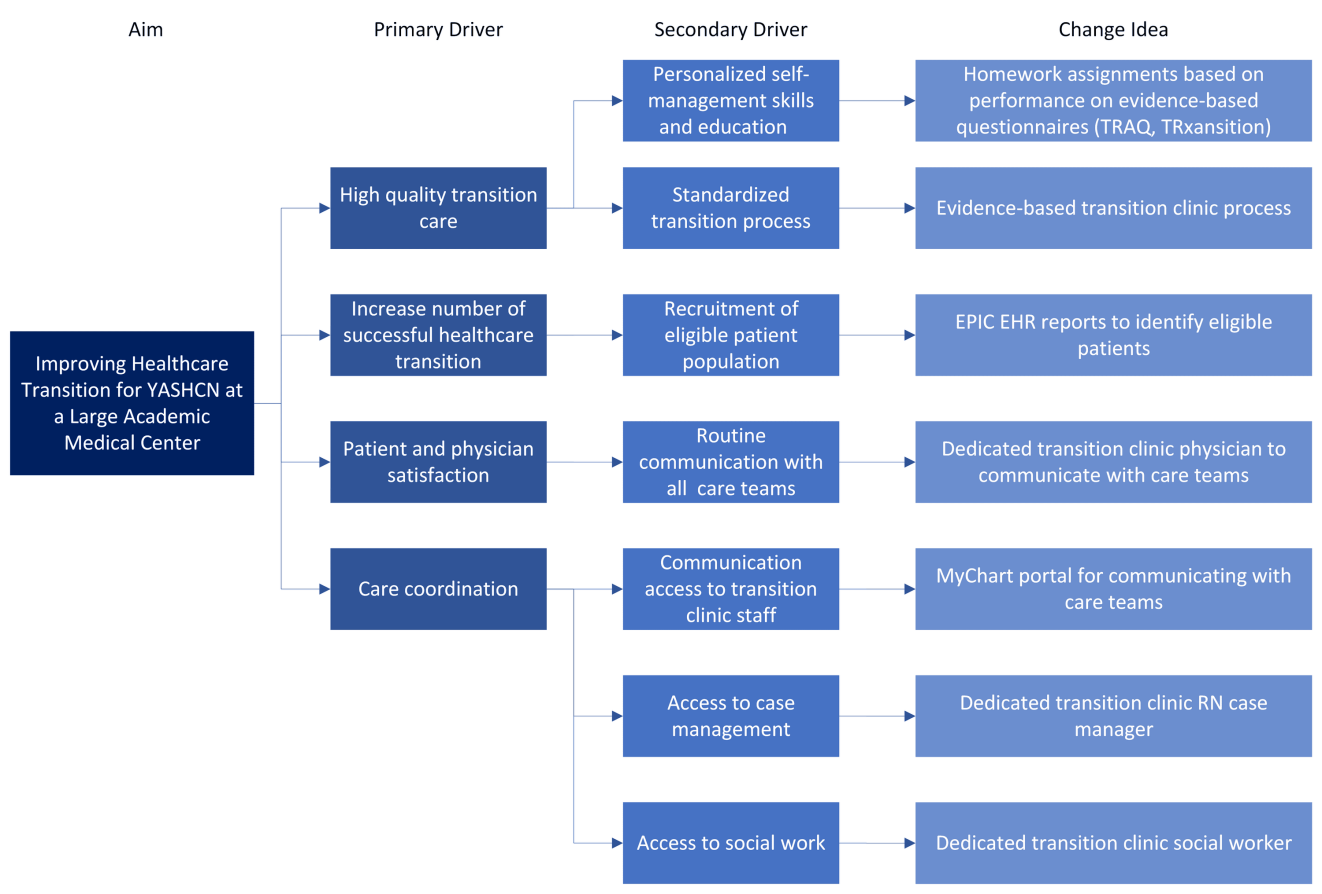
Driver diagram to improving healthcare transition based on physician needs assessment TRAQ: Transition Readiness Assessment Questionnaire; STEER: Supporting Transition Education, Empowerment, and Readiness. STEER is the proposed name for the HCT-based clinic at our institution. Image credit: Ruchita Iyer, MD via Microsoft Visio (Microsoft Corp., USA)

Altogether, these findings reflect the primary and secondary drivers and integral influences for improving HCT at a large academic medical center, as identified in the physician needs assessment (Figure 3). When considered alongside prior literature, these results support incorporating key drivers into the design of a consultative HCT clinic.

The strengths of this project include the mixed-methods design, which allowed quantitative estimates of barrier prevalence and agreement to be contextualized by in-depth qualitative perspectives. The data collection model and plan for reimplementing stakeholder surveys provide an opportunity to collect longitudinal data and assess the long-term impact of the HCT clinic on transition readiness and the integration of transferred YASHCN.

This project was conducted in an academic medical center affiliated with a standalone children’s hospital, a university tertiary/quaternary center, and county hospitals in an urban setting, which may limit generalizability to other healthcare environments. Additional limitations include the subjectivity of qualitative data, the lack of longitudinal outcome data, and the limited ability to assess changes in perceived challenges over time. These one-time interviews provide a snapshot of physician perspectives at a single point in time. Future iterations of this work will expand interviews to include clinic staff, patients, and support persons to complement clinician perspectives and inform ongoing adaptation of policies, resources, and transition services to address unmet needs.

Overall, findings from this physician needs assessment support the development of an evidence-based HCT clinic, highlighting both feasibility and sustainability during the pilot phase and beyond. Next steps include recruiting a dedicated physician medical director, a nurse case manager, and a social worker; defining the eligible pilot patient population; finalizing the clinic location, with consideration of telehealth to enhance accessibility; and implementing a sustainable reimbursement model to ensure long-term viability.

## Conclusions

This mixed-methods needs assessment provides physician-informed, institutionally relevant insight into barriers and opportunities related to HCT for young adults with special healthcare needs. By integrating quantitative estimates of barrier endorsement with qualitative perspectives, this study identifies key areas of consensus among physicians, including the need for improved care coordination, enhanced patient and caregiver preparation for the adult model of care, greater access to adult primary care, and more robust training and clinical guidance specific to the care of young adults with special healthcare needs. These findings highlight structural and educational gaps that may hinder successful transition and continuity of care.

Participating physicians emphasized a collaborative, patient-centered approach to HCT, with proposed solutions focused on strengthening communication between pediatric and adult care teams, standardizing transition processes, and providing dedicated transition services supported by ancillary staff. The alignment between physician-identified barriers and proposed solutions underscores the potential value of a centralized HCT clinic designed to address both system-level inefficiencies and patient-level readiness. These findings establish a strong foundation for developing a comprehensive HCT clinic within a large academic medical center. Future work should prioritize implementing this model, incorporating objective measures of transition readiness and outcomes, and evaluating effectiveness and sustainability over time. Exploration of scalability across diverse healthcare settings will be important to inform broader dissemination and long-term impact.

## Appendices

**Table 5:** Healthcare transition needs assessment interview structure This table presents the structured interview guide used to assess adult clinician perceptions of healthcare transition for young adults with special healthcare needs at UT Southwestern Medical Center. HCT: healthcare transition; YASHCN: young adults with special healthcare needs; SHCN: special healthcare needs; UTSW: University of Texas Southwestern Medical Center

Section	Item	Question / content	Response format
Interview Setup	Consent	Verbal consent for recording interview (Audio/Teams recording).	Verbal confirmation
Objective	Statement	The purpose of this interview is to elicit adult clinician perceptions on the current state of healthcare transition at UT Southwestern Medical Center. Healthcare transition is the process of moving from a pediatric or family-centered model of care to an adult or patient-centered model of care. While all adolescents and young adults benefit from a successful healthcare transition, we know that this process is integral for those young adults with special healthcare needs (YASHCN) as it has been demonstrated in the literature that the transition period is associated with worse health outcomes and higher healthcare utilization by this population.	Scripted statement
Purpose	Statement	Through today’s interview, we seek to discuss the challenges adult clinicians face in assuming care of and providing longitudinal care to the YASHCN patient population. Once we complete the full interview process, we will assess for areas of overlap within the UT Southwestern health system in hopes of developing solutions for this growing patient population and the physicians who care for them. Though many of our faculty also provide care at Parkland, we ask that you only speak to your experience at UT Southwestern, so that we can target UT Southwestern-based solutions. We hope to complete a future iteration of this project focused on the Parkland experience.	Scripted statement
Physician Characteristics	Q1	What is your specialty, and what residency and fellowship training have you completed?	Open-ended
	Q2	How many years have you been in practice (post-residency/fellowship)?	Numeric
YASHCN Definition	Q3	How do you define the population of YASHCN?	Open-ended
	Q4	What subspecialty-specific diseases would you include in this YASHCN patient population?	Open-ended
Transition Challenges	Q5	What challenges do you face within your subspecialty in assuming care of YASHCN (i.e. the first few visits)?	Open-ended
	Q6	What system-wide challenges do you face in assuming care of YASHCN?	Open-ended
	Q7	What challenges do you face within your subspecialty in providing longitudinal care to YASHCN?	Open-ended
	Q8	What system-wide challenges do you face in providing longitudinal care to YASHCN?	Open-ended
Barrier Assessment	Q9a	Difficulty accessing pediatric medical records	Not a barrier / Minor barrier / Major barrier
	Q9b	Poor communication with transferring pediatric providers	Not a barrier / Minor barrier / Major barrier
	Q9c	Lack of access to patient’s adult primary care physician	Not a barrier / Minor barrier / Major barrier
	Q9d	Lack of access to other pediatric or adult subspecialty physicians involved in care	Not a barrier / Minor barrier / Major barrier
	Q9e	Lack of access to needed specialty services (physical/occupational/speech/nutrition services)	Not a barrier / Minor barrier / Major barrier
	Q9f	Time needed to complete the necessary paperwork/forms for patients with SHCN	Not a barrier / Minor barrier / Major barrier
	Q9g	Poor patient or parent preparation for the adult model of care	Not a barrier / Minor barrier / Major barrier
	Q9h	Patient or parent mistrust of new adult providers or the adult healthcare system	Not a barrier / Minor barrier / Major barrier
	Q9i	Not enough social work support	Not a barrier / Minor barrier / Major barrier
	Q9j	Not enough care coordination support	Not a barrier / Minor barrier / Major barrier
	Q9k	My own lack of knowledge about pediatric-onset medical conditions	Not a barrier / Minor barrier / Major barrier
	Q9l	Lack of existing clinical guidelines for the care of young adults with pediatric-onset SHCN	Not a barrier / Minor barrier / Major barrier
	Q9m	Lack of residency/fellowship training in the care of YASHCN	Not a barrier / Minor barrier / Major barrier
Training Exposure	Q10	During your residency training, how much training did you receive related to the care of adults with pediatric-onset SHCN?	None / Not enough / Enough / Too much
	Q11	In training, did you have responsibility for providing care to YASHCN? If so, what settings (inpatient, outpatient, other)? If yes, do you think there was enough exposure in each setting?	Open-ended
	Q12	Did you receive formal curriculum on the care of YASHCN? If yes, please provide an estimated cumulative number of hours.	Open-ended
	Q13	In what ways did your residency training program prepare you to care for patients with pediatric-onset SHCN?	None; <2hrs (eg 1–2 noon conferences); 2–5hrs (eg half-day workshop); >5hrs (eg workshop series or dedicated rotation)
Qualitative Elaboration	Q14	For the prompts you identified as a major barrier, can you describe the specific challenges or clinical scenarios you have faced?	Open-ended
Proposed Solutions	Q15	What proposed solutions do you have to improve your subspecialty’s ability to assume and provide care to YASHCN?	Open-ended
	Q16	What proposed solutions do you have to improve the ability of the UTSW system to assume and provide care to YASHCN?	Open-ended
